# Fused filament fabrication of thermoplastics in high vacuum without convective heat transfer

**DOI:** 10.1038/s41598-025-13181-2

**Published:** 2025-07-28

**Authors:** Marina Kühn-Kauffeldt, Marvin Kühn, Noé Perrin, Wolfgang Saur

**Affiliations:** 1 Institute for Electrical Energy Systems, University of the Bundeswehr Munich, Werner-Heisenberg-Weg 39, 85577 Neubiberg, Germany; 2 CentraleSupélec, 3 Rue Joliot Curie, Gif-sur-Yvette, 91190 France; 3 Chair of Construction Materials, University of the Bundeswehr Munich, Werner- Heisenberg-Weg 39, 85577 Neubiberg, Germany

**Keywords:** High vacuum, Fused filament fabrication, Convective heat transfer, Mechanical properties, PLA, Mechanical engineering, Techniques and instrumentation

## Abstract

In-space additive manufacturing (AM) offers significant potential to expand human space exploration beyond low Earth orbit and the moon. Although extrusion-based AM has proven feasible in zero gravity, the functionality of such a process in orbit-like vacuum conditions with practically no convective heat loss remains barely explored. To this end, a Fused Filament Fabrication (FFF) system was designed that successfully operated in high vacuum at 10^− 4^ mbar where convective heat transfer is negligible. Polylactic acid (PLA) tensile specimens were fabricated in three orthogonal print orientations under high vacuum conditions. Tensile testing, scanning electron microscopy, and micro-computed tomography were employed to assess tensile strength, elongation at break, void content, and the effects of vacuum on thermal dissipation. The objective was to evaluate how the vacuum environment influences the anisotropy of mechanical properties in printed parts. The absence of convective losses improved the layer bonding strength of specimens printed in the vertical (z) direction with load applied perpendicular to the filament strand orientation (V90), compared to specimens printed in horizontal (x or y) direction with load applied perpendicular to filament strand orientation (H90). This was attributed to the considerably slower cooling process in the case of V90 specimens. Moreover, thermal insulation provided by the vacuum environment had a beneficial influence on heat break and hot end. In vacuum, the set extrusion temperature was closer to the temperature measured at the tip of the nozzle. This study also identifies a temperature undershoot during decrease in hot end temperature in the printing process, which adversely affected interlayer adhesion. The findings indicate that the current PID-based hot-end control requires optimization to enhance the temperature control rate and minimize thermal deviations. These insights contribute to a deeper understanding of thermoplastic processing under convection-free conditions using fused filament fabrication (FFF).

**Supplementary Information:**

The online version contains supplementary material available at 10.1038/s41598-025-13181-2.

## Introduction

In-space additive manufacturing (AM) is a promising approach to resolve existing limitations to manned spaceflight beyond low Earth orbit and the Moon, particularly for weight- or volume-restricted payloads^1^. To overcome these challenges, AM has been proposed to fabricate miniaturized satellites^2^ and replacement parts, as well as to leverage the potential to manufacture large-scale structures in orbit and surgical tools, enable medical care on long-term missions^3,4^.

Fused filament fabrication (FFF) is among the most widely adopted AM techniques^5^. Various projects have orbited FFF systems on the International Space Station (ISS) and proven the general functionality of FFF in a microgravity environment^6–9^. Besides the radiative environment and extreme temperatures, the feasibility of FFF in open space still has to be verified. In this context, operation at vacuum pressures equivalent to space conditions is a key requirement. For example, in low Earth orbit, where off-Earth manufacturing activities are planned by space agencies^10^, the pressure is about 10^− 7^ to 10^− 9^ mbar. Although the high vacuum range (10⁻³ to 10⁻⁸ mbar), as defined by ISO 3529^11^, does not fully encompass the ultra-high vacuum conditions characteristic of space, it nevertheless results in the suppression of convective heat transfer due to the significantly reduced gas particle density. This reduction in convective effects is anticipated to have a substantial impact on the FFF process, particularly by altering the thermal environment around the filament strand. The lack of convective cooling may influence filament solidification dynamics, interlayer adhesion, and overall part quality, making thermal management a critical consideration in vacuum-based additive manufacturing.

On Earth, such pressure conditions can be replicated in a vacuum chamber. Given a chamber with a volume of 0.027 m³ and filled with air at room temperature, a pressure level of 10^− 3^ mbar and below will result in a molecular flow regime for gases (Kn > 0.5)^12^. In this scenario, the mean free path of the gas particles is at least in the range of the dimensions of the chamber. Hence, their collision frequency is low. As a result, convective heat transfer becomes negligible—approaching conditions similar to those in low Earth orbit. Under such reduced-pressure environments, the diminished thermal dissipation alters the heat transfer balance during material extrusion. This potentially affects filament cooling rates, interlayer bonding, and residual stress development. Therefore, starting from this pressure regime, notable deviations in FFF process behavior compared to atmospheric conditions are expected, driven by the fundamental shift from convective to primarily radiative and conductive heat transfer mechanisms.

Quinn et al. adapted a commercially available FFF system to work in low vacuum (10 mbar)^13^. Matignly et al. have developed a vacuum assisted extrusion unit for large-format additive manufacturing and demonstrated significant pore reduction during acrylonitrile butadiene styrene (ABS) extrusion at the pressure of 160 mbar^14^. Influence of vacuum in the range 1 mbar on printing quality of Polyetherehterketone (PEEK) specimens compared to those produced at atmospheric pressure was studied Liu et al.^15^ while Wang et al. has developed a model predicting the mechanical properties of specimens produced at 1 mbar^16^. However, only Spicer et al. have demonstrated the extrusion of materials such as polylactic acid (PLA), polyethylene terephthalate glycol (PETG), ABS, polyetherketoneketone (PEKK), and polyetherimide (PEI) at pressures as low as 10⁻⁵ mbar using a passively cooled hot end design^17,18^, when using a passively cooled hot end design. Nonetheless, the specific effects of high-vacuum conditions (< 10^− 3^ mbar) on the microstructural characteristics, thermal transport behavior, and mechanical properties of FFF-printed specimens remain largely unexplored in prior vacuum-based FFF research.

The absence of convective heat transfer will strongly influence heat dissipation processes, both on the printer hardware level, such as motors and the hot end, as well as the fundamental melt solidification process of the extruded polymer.

In the FFF process, thermal energy is used to melt polymers. Here the hot end is responsible for the local energy input that melts the polymer. The heat break inhibits heat transfer to the upper, solid portion of the filament, which must remain rigid to ensure stable and controlled extrusion. In this work, the heating and extrusion were performed under vacuum, which changed the heat transfer compared to atmospheric conditions. This, in turn, leads to the necessity of ensuring proper machine cooling as well as the consideration of an altered cool-down characteristics of the printed polymer parts. Therefore, this work aims to investigate the effects of strongly reduced convective heat losses in high vacuum on the extrusion process itself.

To this end, an FFF system with in situ thermal monitoring capable of operating in a high vacuum environment was designed and built. The parts fabricated under such conditions were assessed via tensile testing, scanning electron microscopy, and computed tomography. The findings of this work contribute to a better understanding the FFF process in convection free vacuum environments.

## Materials and methods

### FFF-system

**Fig. 1:**
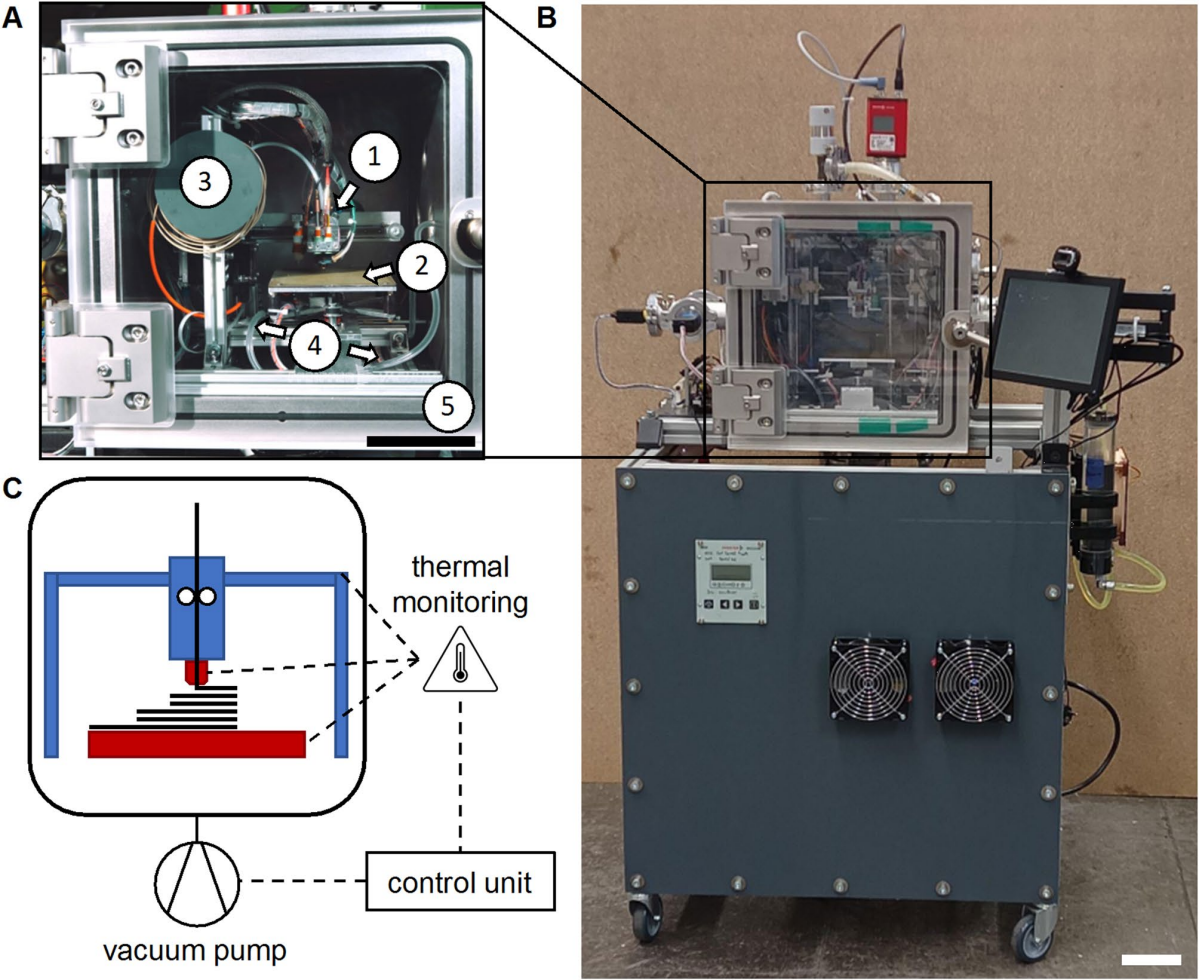
In-house developed FFF-system for operation in vacuum. (**A**) Cartesian FFF-system with (1) the print head on the X and Z-axis, (2) the print bed on the Y-axis, and (3) the filament spool. All motors and the extruder were water cooled via a (4) tubing system connected to a radiator. The FFF-system was positioned inside (5) a vacuum chamber. (**B**) Test stand with the vacuum chamber on top of an alumina frame housing which hosts the vacuum pumps. (**C**) Schematic of the vacuum FFF-system, where a control unit allowed thermal monitoring of the nozzle, the bed, and the water-cooled components (scale bars 10 cm).

Specimens were fabricated using a custom-built FFF system with a build envelope of 100 × 100 × 50 mm^3^ (Fig. 1A). The system uses a belt-driven rectilinear Cartesian kinematic design. The advantage of this system is its simplicity, while moderate printing speed is sufficient due to slow heat dissipation in vacuum. The system was powered by NEMA 17 vacuum-rated motors (4118 M-06P, Lin Engineering, USA). The kinematics featured a Z-axis assembly that supported a horizontally mounted X-axis, allowing vertical movement of the entire X-axis. This configuration reduces the number of potential contamination sources within the vacuum. An E3D Titan Aqua (12 V, 1.75 mm filament, 30 W heater cartridge, UK) together with 450 °C thermistor (Slice Engineering, USA) served as a water-cooled extruder and hot end assembly. Here a full metal hot end was chosen to be able to print a high range of temperatures. Lubricant-free mechanical components were selected to minimize outgassing in vacuum. All motors and the extruder were actively water-cooled (DC12-220 12 V Pump, Phobya, combined with a 120 mm radiator (Alphacool, Germany). A standard 0.4 mm brass nozzle was used for all prints. It was selected for this study due to its high thermal conductivity, which ensures efficient and uniform heat transfer, ensuring smooth and stable PLA extrusion. Within the temperature range required for PLA processing (~ 180–220 °C), the thermal expansion of brass remains sufficiently low to prevent mechanical instability. Given the relatively low abrasiveness of PLA, nozzle wear is minimal, making brass a suitable and cost-effective choice. However, for applications involving higher processing temperatures or abrasive composite filaments, alternative materials such as tungsten carbide may offer enhanced durability and thermal stability.

The print bed was equipped with a Kapton heater (12 V, 48 W, TRU COMPONENTS, Germany). The hardware was controlled by the RUMBA32 control board (Aus3D, Australia) featuring with SilentStepStic motor drivers (Watterott, Germany). This board offers a wide range of electric ports, which can be used to directly control the vacuum infrastructure and can be operated with an open-source firmware such as Marlin. In theory, the electrical components can operate in vacuum with additional cooling. However, this has not yet been experimentally verified. A BLTouch (Antctlabs, South Korea) served as the bed levelling sensor. The controller ran on Marlin 2.0.7.2 firmware.

### Vacuum setup

The FFF system operated inside a stainless-steel vacuum chamber (inner dimensions 300 mm x 300 mm x 300 mm) equipped with an acrylic door as a viewport (Fig. 1B). For vacuum generation, a HiPace 80 turbopump, together with a Duo 5 m backing pump and a DCU02 control unit, were used (Pfeiffer Vacuum, Germany). The pressure was monitored via a PKR 251 wide range-pressure-sensor (Pfeiffer Vacuum, Germany). The FFF hardware was electrically connected via standard vacuum-rated electrical feedthroughs to the electronic boards outside the vacuum chamber. For the water-cooling circuit, standard liquid cooling feedthroughs were used to link the serially connected heat sinks to a radiator. This design ensured leak-tight, low-outgassing connections between the heat sinks, water-guiding tubes, and feedthroughs could be ensured, while preserving the necessary flexibility of the kinematic system. The vacuum setup including the FFF system, achieved a base pressure of 10⁻⁵ mbar without operating the extruder unit.

### Vacuum FFF characterisation

To characterize the printing conditions under vacuum, the chamber pressure was recorded during the heating of the hot end and print bed to their respective maximum temperatures. Simultaneously, temperature data from both components were logged via Marlin firmware using internal temperature sensors. PID tuning was conducted using the firmware’s built-in autotuning routine (G-code command M303).

For in situ thermal monitoring, a thermal camera sensor Lepton 3.5 (Teledyne FLIR, USA) together with a PureThermal 2 - Lepton Smart I/O board (Teledyne FLIR, USA) was placed inside the vacuum chamber to monitor nozzle temperature during operation under both atmospheric and vacuum conditions (Fig. 1C). The sensor delivers factory-calibrated radiometric temperature data with a precision of 5%. Sensor temperature data were validated using thermocouple measurements during hot end operation at atmospheric pressure. As only relative comparison of the thermal images (atmosphere vs. vacuum) is performed, no emissivity or reflection calibration was required.

### Specimen fabrication

Filaments with a diameter of 1.75 mm, made from natural (unpigmented) polylactic acid (PLA Natur, Material4Print, Germany) were used without further modification. The G-code was generated using the PrusaSlicer software package (Version 2.4.2, developed in the Czech Republic). The FFF process parameters used in this study are summarized in Table 1.

For all prints, the print bed was covered with a polyethylene terephthalat (PET) foil, which was renewed for every print. This ensured a consistent printing surface and minimized the risk of first-layer delamination or related defects. Subsequently, the vacuum chamber was evacuated using a two-stage pumping system. Each print was started at a chamber pressure of 5 × 10⁻⁴ mbar. The print head movement speed and temperature were adjusted for the first layer to improve bed adhesion. The addition of a brim further enhanced adhesion. After printing, the chamber was vented, and the parts were extracted from the bed.

**Table 1:** FFF process parameters for printing PLA in vacuum. H0 and H90 specimens were printed in horizontal (x or y) direction with load applied parallel (0) and perpendicular (90) to filament strand orientation; V90 specimens were printed in vertical (z) direction with load applied perpendicular to the filament strand orientation.

Parameter	Setting	
Operating pressure [mbar]	~ 10^− 4^	
Nozzle temperature [°C] First layer [°C]	180 200	
Bed temperature [°C]	60	
Chamber temperature [°C]	Room temperature, no additional chamber heating applied	
	*H0 and H90*	*V90*
Nozzle speed [mm/s]	30	10
First layer speed [mm/s]	5	5
Outer perimeter speed [mm/s]	Not applicable	5
Layer height [mm]	0.2	
Extrusion width [mm]	0.45	
Infill pattern	Aligned rectilinear	
Infill density [%]	100	

**Fig. 2:**
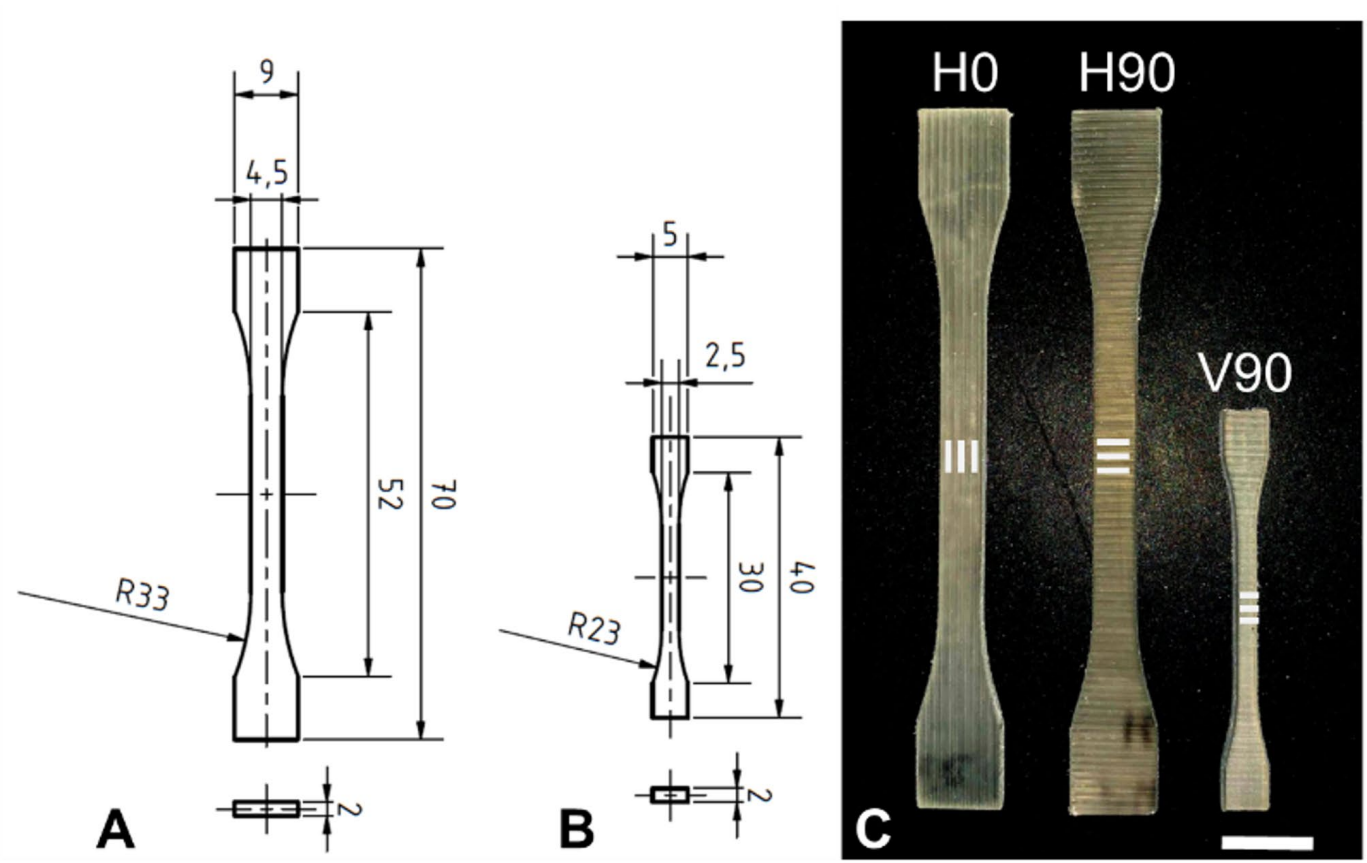
Technical drawing of the H0/H90 tensile bars (**A**) and the V90 tensile bars (**B**). Both designs are scaled versions of the 1BA specimen from DIN EN ISO 527. (**C**) Water-jetted specimens ready for tensile testing. The white lines indicate the main deposition direction. Horizontal specimens were printed in way that the main deposition direction is parallel (H0) or perpendicular (H90) to the loading direction. Vertically oriented specimens (V90) were fabricated to investigate the interlayer bonding with deposition direction perpendicular to loading direction (scale bar 10 mm).

The specimen design enabled testing of tensile characteristics in three orthogonal printing directions (Fig. 2C). Due to reduced heat dissipation in the vacuum environment, vertically oriented specimens (V90) could not be printed as monolithic structures. Instead, rectangular hollow shells (30 mm × 30 mm × 50 mm, wall thickness 2 mm) were fabricated, from which V90 tensile specimens were subsequently extracted using water-jet cutting. Therefore, all specimen geometries were produced using a consistent manufacturing approach to eliminate variability in tensile test results due to differing fabrication methods. Consequently, rectangular plates (80 mm x 45 mm x 2 mm) with unidirectional deposition paths were printed, from which the tensile bars were water jetted to meet the dimensional specifications of DIN EN ISO 527. Specimens printed as horizontal plates with deposition paths parallel to the loading direction are referred to as H0. In contrast, horizontal specimens with deposition paths orthogonal to the loading direction are referred to as H90. All tensile bars were scaled versions of the design 1BA (Fig. 2A and B), adjusted for the limited build envelope of the in-house-developed FFF system.

### Tensile testing

To assess the effects of high vacuum on the mechanical quality of FFF parts, tensile bars in three orthogonal directions were fabricated: (i) H0, horizontally printed tensile bars, where the deposited filament strands are parallel to the loading direction, (ii) H90, horizontally printed tensile bars, with the main deposition direction perpendicular to the loading direction, and (iii) V90, vertically printed tensile bars, where the layer direction is perpendicular to the loading direction.

Tensile tests (*n* = 7) were conducted on each specimen type on a Zwickline Z2.5 (ZwickRoell, Germany) equipped with a 2.5 kN load cell at a crosshead speed of 10 mm/min. Young’s modulus was determined from the linear portion (0.5 to 2.5% strain) of the force-strain curve.

### Specimen imaging

The fracture surface was analyzed with scanning electron microscopy (SEM, EVO LS15, Zeiss, Germany) in high vacuum mode at an acceleration voltage of 20 kV. Before SEM observations, a ~ 10 nm gold layer was sputter-coated onto all specimen surfaces.

The SEM images were analyzed using ImageJ software (version 1.54p) to quantify void size. Void areas were manually identified, and their size was evaluated as a function of the printed layer’s distance from the print bed (Fig. S1).

To further investigate interlayer bonding and morphology along the z-direction, micro-computed tomography (µCT; Bruker SkyScan 1172, USA) was performed on unloaded fracture surfaces of H90 and V90 tensile specimens. Scans were conducted at a source voltage of 43 kV, 20 µA current and a spatial resolution of 10 μm. Image reconstruction was performed using NRecon software (version 1.7.4.6, Bruker SkyScan), and 3D porosity was analyzed using global thresholding CTAn (version 1.18.8, Bruker SkyScan).

### Statistical analysis

Data were statistically analyzed using Prism Prism 9.2.0 (GraphPad Software, USA). After confirmation of normal distribution, two-way ANOVA with *post hoc* multiple comparisons was applied. Values are reported as mean ± standard deviation. ****p ≤ 0.0001, ***0.0001 < *p* ≤ 0.001, **0.001 < *p* ≤ 0.01, and *0.01 < *p* ≤ 0.05 were used to indicate the level of significance.

## Results and discussion

**Fig. 3:**
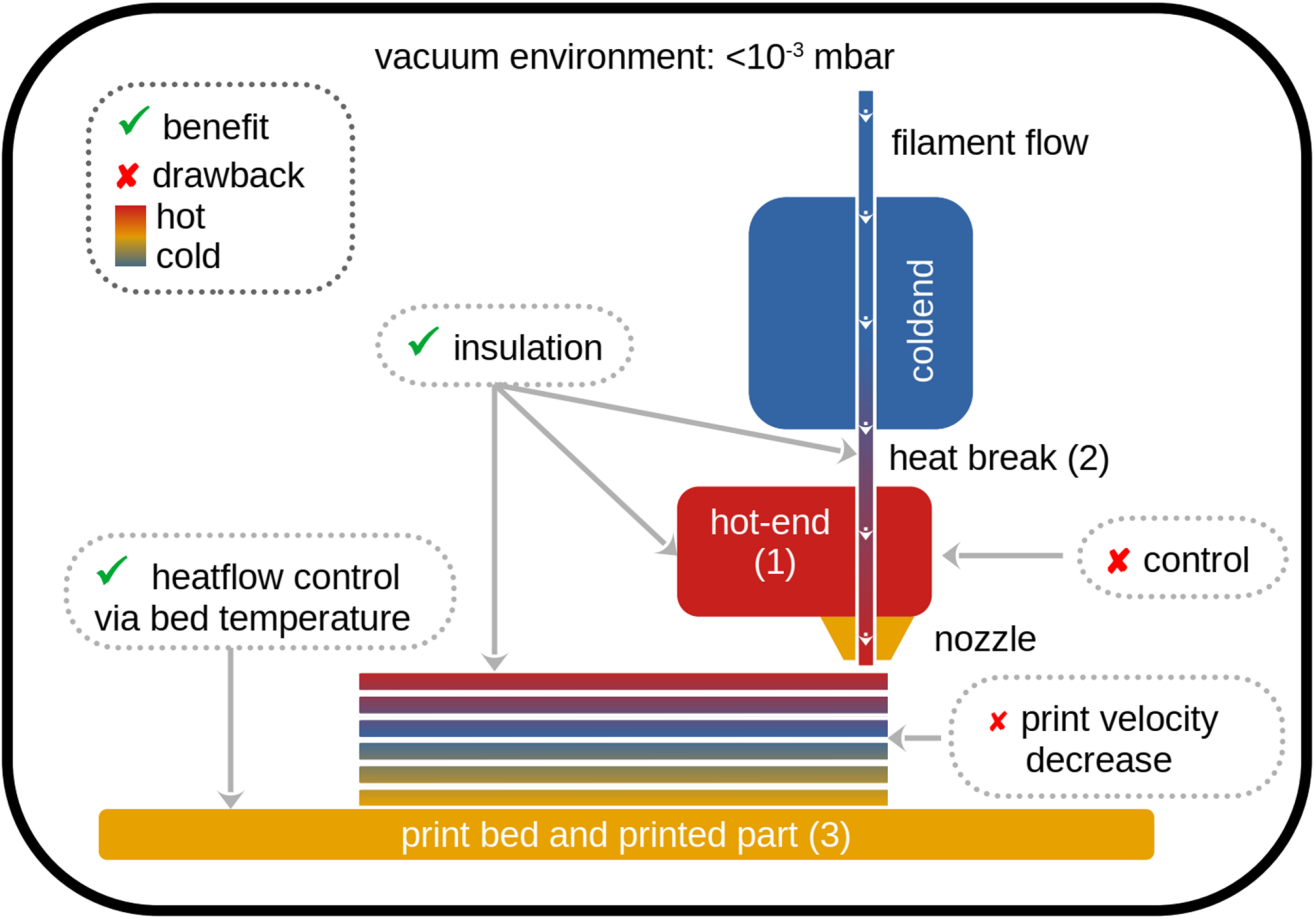
Schematic (not to scale) of the FFF process in vacuum with 3 different zones impacted by the vacuum environment: (1) the hot-end (2) the heat-break and (3) the printed part. ✓ represents beneficial effects, while × indicates drawbacks.

This study presents a qualitative evaluation of how a vacuum environment influences the Fused Filament Fabrication (FFF) process. To illustrate these effects, Fig. 3 outlines three key zones within the system that are distinctly impacted by vacuum conditions: (1) the hot end, which experiences reduced heat conduction but exhibits delayed temperature regulation; (2) the heat break, which similarly benefits from reduced thermal conduction; and (3) the printed part, where the absence of convective cooling necessitates substantially reduced print velocities and relies exclusively on conduction and radiation for heat dissipation. The observed effects within each zone are analyzed and discussed in the following sections.

### High vacuum compatible FFF-system

When operating the high vacuum chamber with a passive FFF-system inside, a base pressure of 5 × 10⁻⁵ mbar was achieved, where a molecular gas flow regime prevails^12^. Both the chamber pressure and power consumption of the rotary vacuum pump (proportional to gas load) were monitored during operation. No measurable increase in power consumption or pressure was detected during water-cooling operation or mechanical movement.

**Fig. 4:**
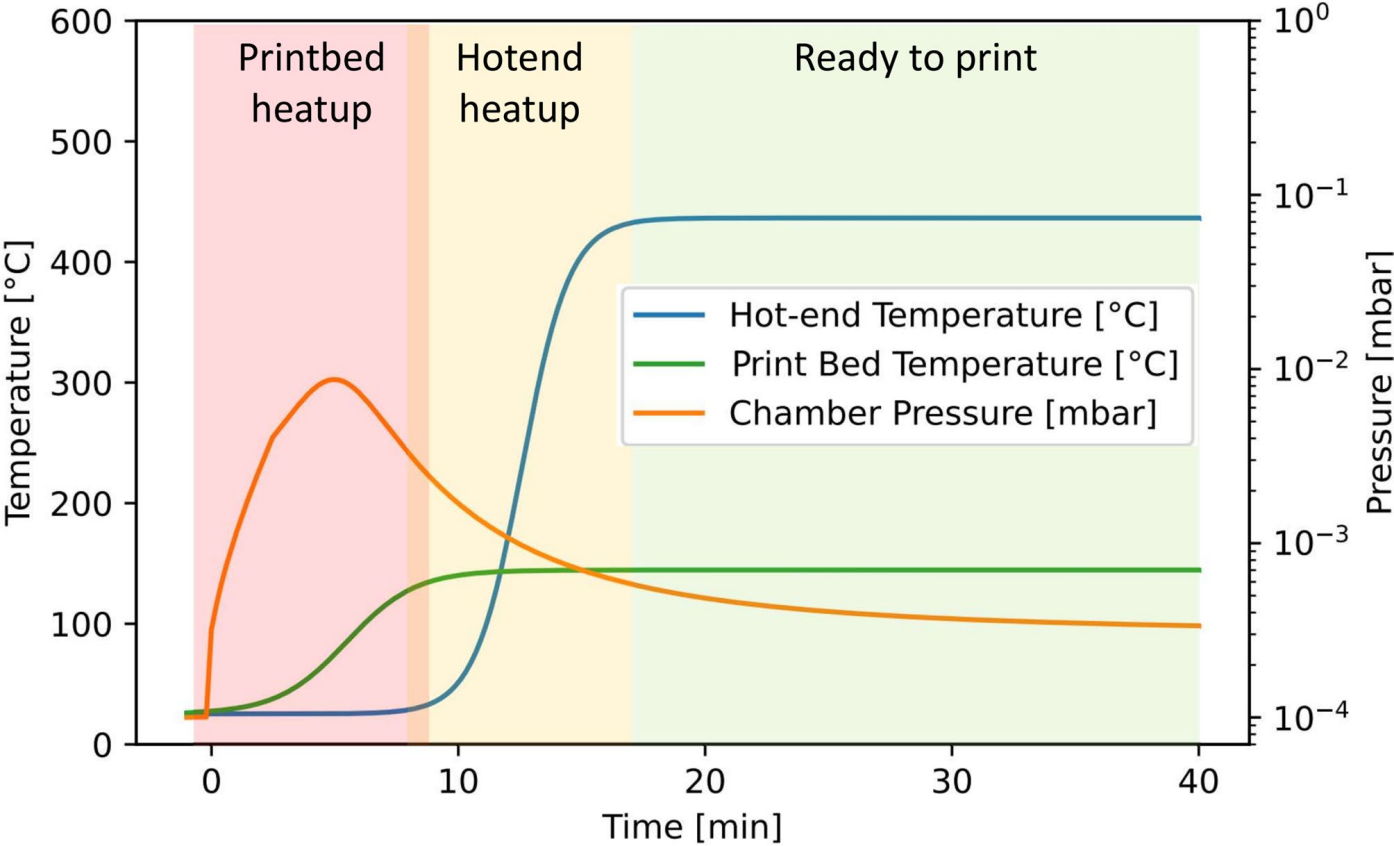
Chamber pressure fluctuation (orange) relative to the bed temperature (green) and hot-end temperature (blue) are shown for a high temperature polymer printing process in a high vacuum. The red, yellow and green areas mark different phases of the printing procedure.

Figure 4 illustrates a typical pressure curve recorded during different printing phases. Starting from the base pressure of 1 × 10^− 4^ mbar, a short pressure rise can be observed during print bed heat-up (red area) with a peak at t = 4 min at around 80 °C, after which the pressure quickly drops again towards the base pressure. The pressure rise resulted from evaporation of moisture and adsorbed gases, previously condensed on the print bed surface during venting. The hot-end heat-up (yellow area) did not significantly increase chamber pressure. This is attributed to the smaller surface area of the hot end. The pumping unit maintained a stable pressure level of 3 × 10⁻⁴ mbar (green area), which partially mitigated vacuum contamination; however, the potential influence of residual gas species cannot be fully excluded. The difference in the base pressure probably originated from the outgassing of the heated polymer melt during the printing process itself. Despite polymer outgassing, pump capacity maintained high vacuum and prevented convective heat transfer. The active water-cooling system effectively prevented component overheating.

The heat transfer phenomena in high vacuum led to the necessity of adapting the PID parameters for the thermal control of the hot-end. Via a PID parameter tuning cycle in vacuum the optimal parameters resulted in *P* = 9.74, I = 0.31, and D = 75.37. These values avoided temperature deviation (± 5 °C) while ensuring reasonably fast heat-up. For atmospheric printing, PID values were *P* = 26.46, I = 2.41, D = 72.51. The P value represents the proportional term of the feedback control. It is a more than two-fold increase for the atmospheric process compared to operation in vacuum. The increased P value reflects convective heat loss in atmosphere, absent in vacuum. The elevated I value compensates for temperature deviations due to thermal losses. The elevated I value compensates for temperature deviations due to thermal losses. The D term controls the rate of change and damping during hot-end heating, which is geometry-dependent and therefore similar under both conditions^19^.

**Fig. 5:**
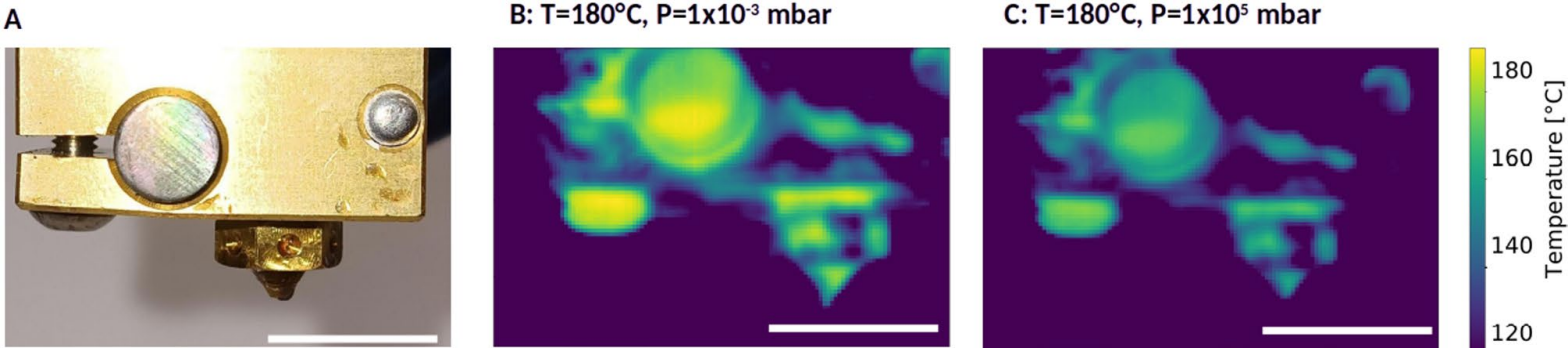
Photographic view of the hot-end (**A**) as recorded as thermographic images by the thermal camera in (**B**) vacuum and in (**C**) atmospheric pressure conditions at a set temperature of 180 °C. The thermographic images clearly indicate the temperature reductions due to convective heat losses when operating at atmospheric conditions (scale bars 10 mm).

Hot-end thermal behavior in vacuum was evaluated using a thermal camera mounted on the print head. As is exemplified in Fig. 5 for a set temperature of 180 °C, the measured hot-end temperature differed significantly between vacuum and atmospheric conditions. In vacuum, the nozzle temperature reaches 179 °C, and the maximal measured temperature in the image is 189 °C (located on the screw left to the nozzle). In regular atmospheric pressure conditions, however, the nozzle surface reached approximately 161 °C, with the maximal measured temperature in the image being 177 °C (detected on the screw left to the nozzle). Hence, a temperature difference of at least 10 °C can be assumed in this case.

**Fig. 6:**
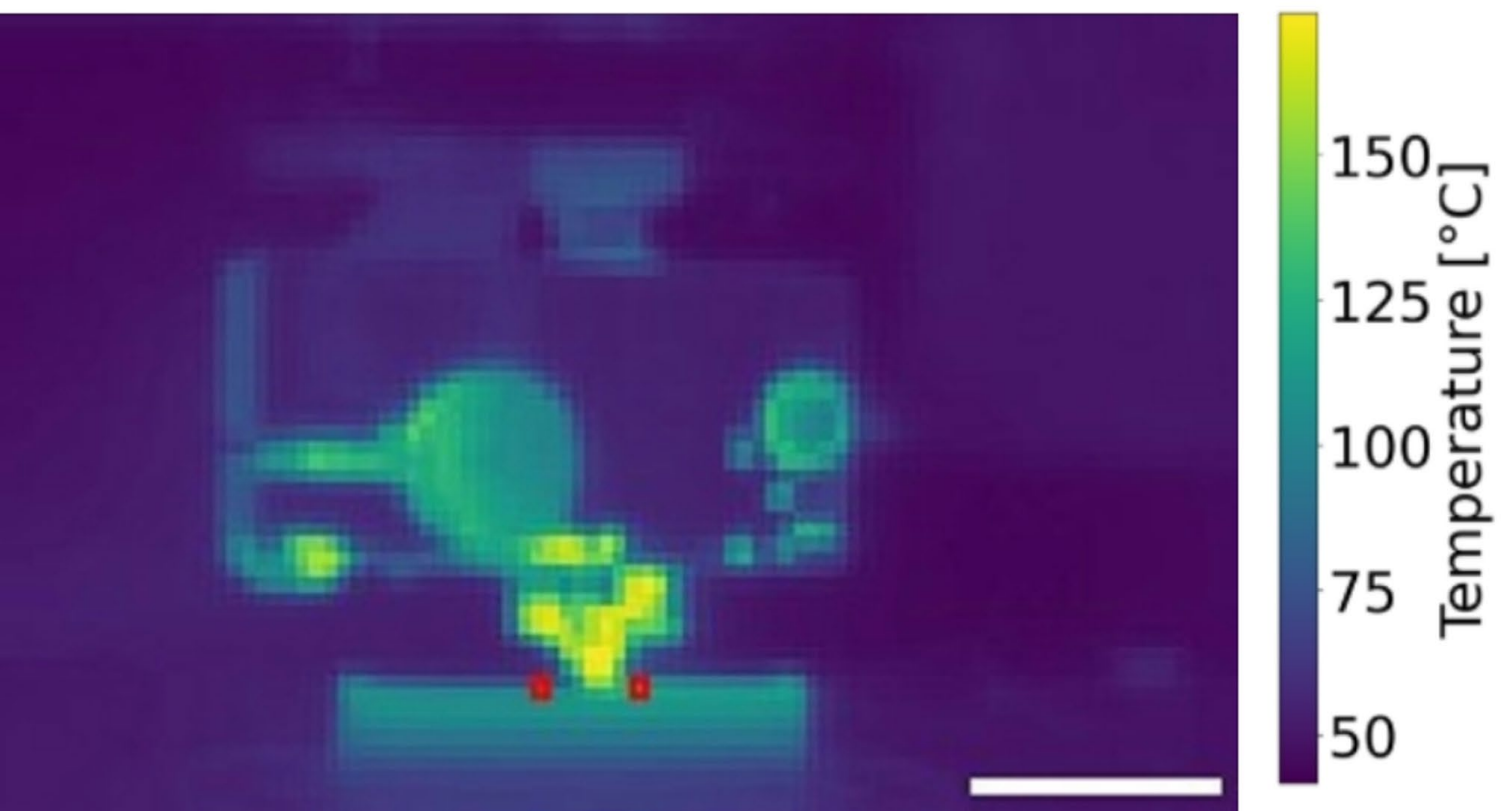
Example of thermographic image of a test print. Here a 2 mm thick wall was printed at 180 °C set temperature at 10 mm/s printing speed on air and in high vacuum. The red marks indicate the position of filament temperature evaluation (scale bar 10 mm).

Additionally, filament surface temperatures during wall-printing at 180 °C were compared in air and vacuum. Here the thermal detector was mounted to move together with the nozzle, allowing temperature tracking 2 mm before and after nozzle deposition (Fig. 6). An average filament temperature of 132 °C was recorded in vacuum, compared to 121 °C in air.

Considering the previously discussed PID control precision, the observed relative temperature difference in the hot-end between vacuum and atmospheric conditions may be attributed not only to convective heat losses in air but also to inherent fluctuations in controller performance, which can vary within a range of ± 5 °C.

### Tensile testing and structural effects

The influence of the vacuum environment on the printed parts became evident when comparing process parameters for different geometries. While the H0 and H90 specimens were successfully printed at 30 mm/s, V90 specimens required a significantly slower speed of 10 mm/s. The printing speed of the outer perimeter of the rectangular tower, from which the V90 specimens were cut out, was further reduced to 5 mm/s to maintain surface quality. This adjustment was necessary due to different part geometries and differing cooling mechanisms. While convective cooling is entirely absent under vacuum, printed parts can only cool via conduction and radiation. The heat dissipation via conduction from the printed part towards the print bed was more efficient for the H0 and H90 specimens compared to V90 ones. As the H0 and H90 specimens were printed as flat rectangular geometry on the bed surface, their contact area to the bed was 15 times larger. Their heights were 25 times lower than it was the case for V90 specimens, which improved conductive coupling to the print bed. Thus, bed conduction dominated their cooling. For the V90 specimen, however, the conductive cooling was much slower due to a smaller bed contact area and a significantly greater height of the specimen. Although radiative cooling might have a higher contribution in the V90 specimens, the temperature gradient was insufficient to compensate for the reduced thermal conduction, leading to a significantly reduced printing velocity.

The effect of high vacuum conditions on heat-break insulation became evident during the fabrication of V90 specimens. Extrusion failure is common with stainless-steel heat breaks and PLA under atmospheric conditions. This issue is typically due to heat creep, where heat propagates from the hot end upward, softening the filament before extrusion and disrupting the piston-like motion. In contrast, V90 printing in vacuum—even over durations exceeding 4 h—resulted in no extrusion issues. At atmospheric conditions, using the same print speed and a low extrusion temperature (T_air_ = 200 °C), failures consistently occurred after 2–3 h unless the speed was significantly increased. These results suggest that vacuum enhances heat-break insulation, enabling stable, long-duration extrusion at low speeds.

**Fig. 7:**
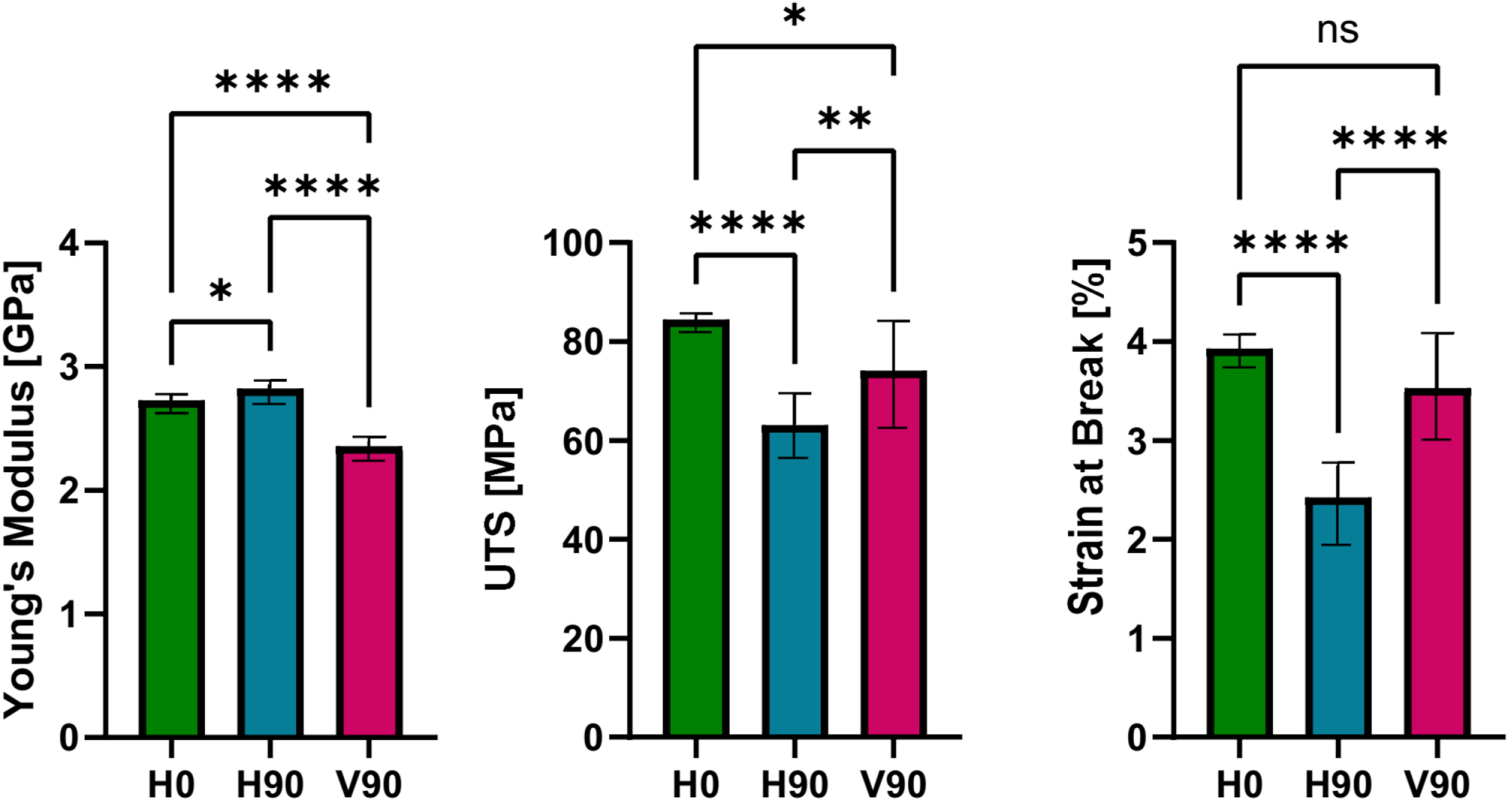
Results of the Young’s modulus, ultimate tensile strength and elongation at break, for three different tensile bar orientations (H0 green, H90 blue, V90 purple). ****p ≤ 0.0001, ***0.0001 < *p* ≤ 0.001, **0.001 < *p* ≤ 0.01, and *0.01 < *p* ≤ 0.05 were used to indicate the level of significance.

Moreover, the influence of the vacuum on the FFF process becomes evident, when investigating mechanical properties of printed specimen. For H0 specimens no significant differences in ultimate tensile strength (UTS) and elongation at break (Fig. 7) compared to values reported in the filament datasheet were stated^20^. Since these specimens were loaded along the filament direction, the bulk polymer properties governed the mechanical response^21^.

The situation differs for H90 and V90 specimens, where filament strands are oriented perpendicular to the loading axis. In these configurations, interlayer bonding becomes a key factor^22^. Contrary to prior studies - where V90 specimens exhibited the lowest ultimate tensile strength (UTS) when printed in air^21^ - the present study in vacuum shows that the lowest UTS values were obtained for the H90 specimens (Fig. 7). This outcome may be attributed to differences in cooling dynamics under vacuum conditions. Specifically, it is hypothesized that the H90 specimens experienced a more rapid cooling process compared to the V90 specimens, leading to comparatively weaker interlayer adhesion in the former and improved bonding in the latter. To validate this hypothesis, future studies should incorporate interlayer temperature measurements, enabling the calculation of healing degree and assessment of interlayer bonding quality^23^.

**Fig. 8:**
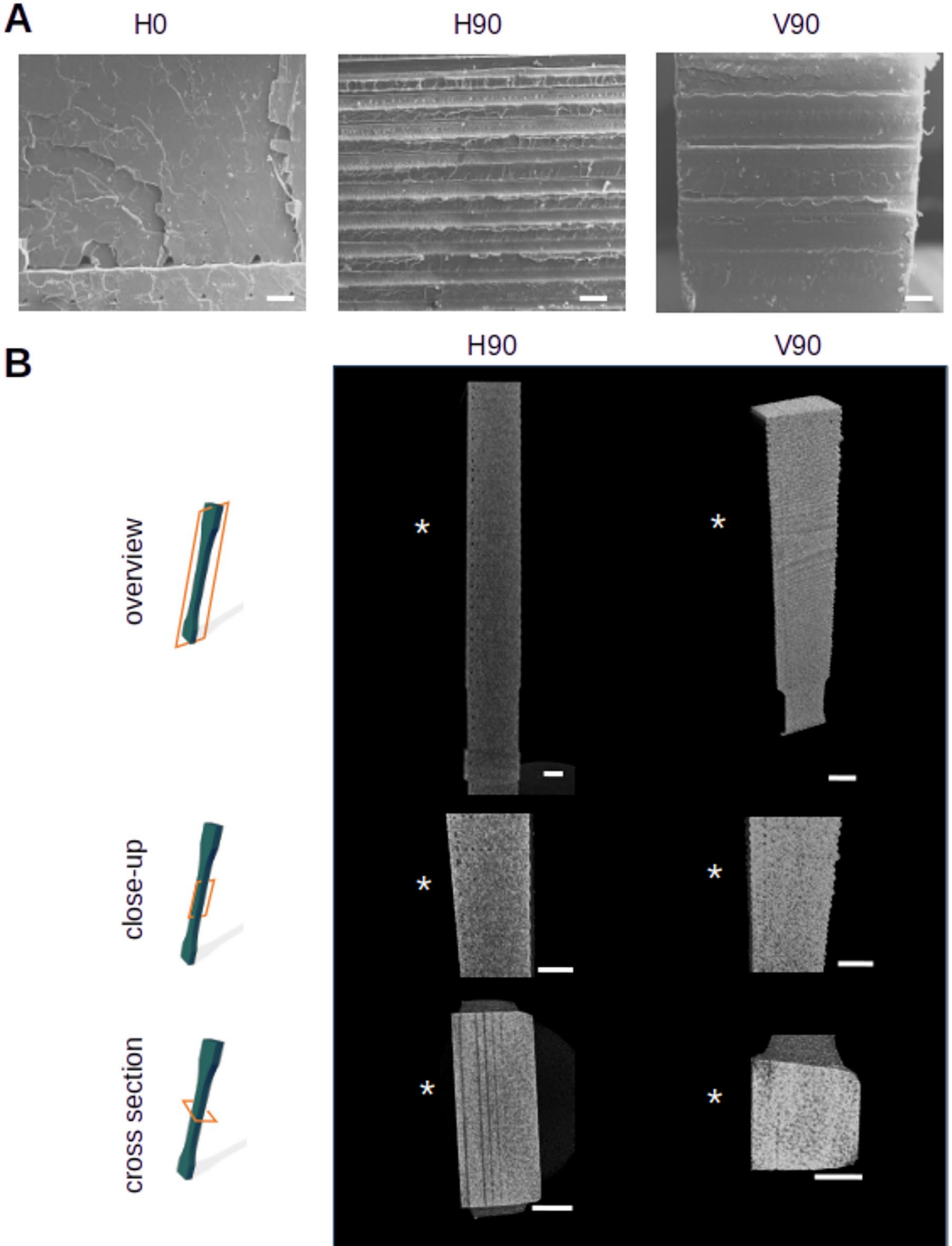
Fracture surfaces and internal structures of the tensile bars after testing. (**A**) SEM micrographs of the fracture surfaces of the tensile bars show a homogeneous texture without voids with exception of the first layers of the H0 specimen in vacuum. The H90 specimens exhibit a fracture pattern with a clearly layered structured (scale bars 200 μm). (**B**) µCT scan reconstructions of the tensile bars highlight the presence of voids depending on the printing direction of the tensile bars (scale bars 1 mm). *Indicates side of print bed for H90 specimens and outer perimeter layer printed at reduced speed for V90 specimens, respectively.

**Fig. 9:**
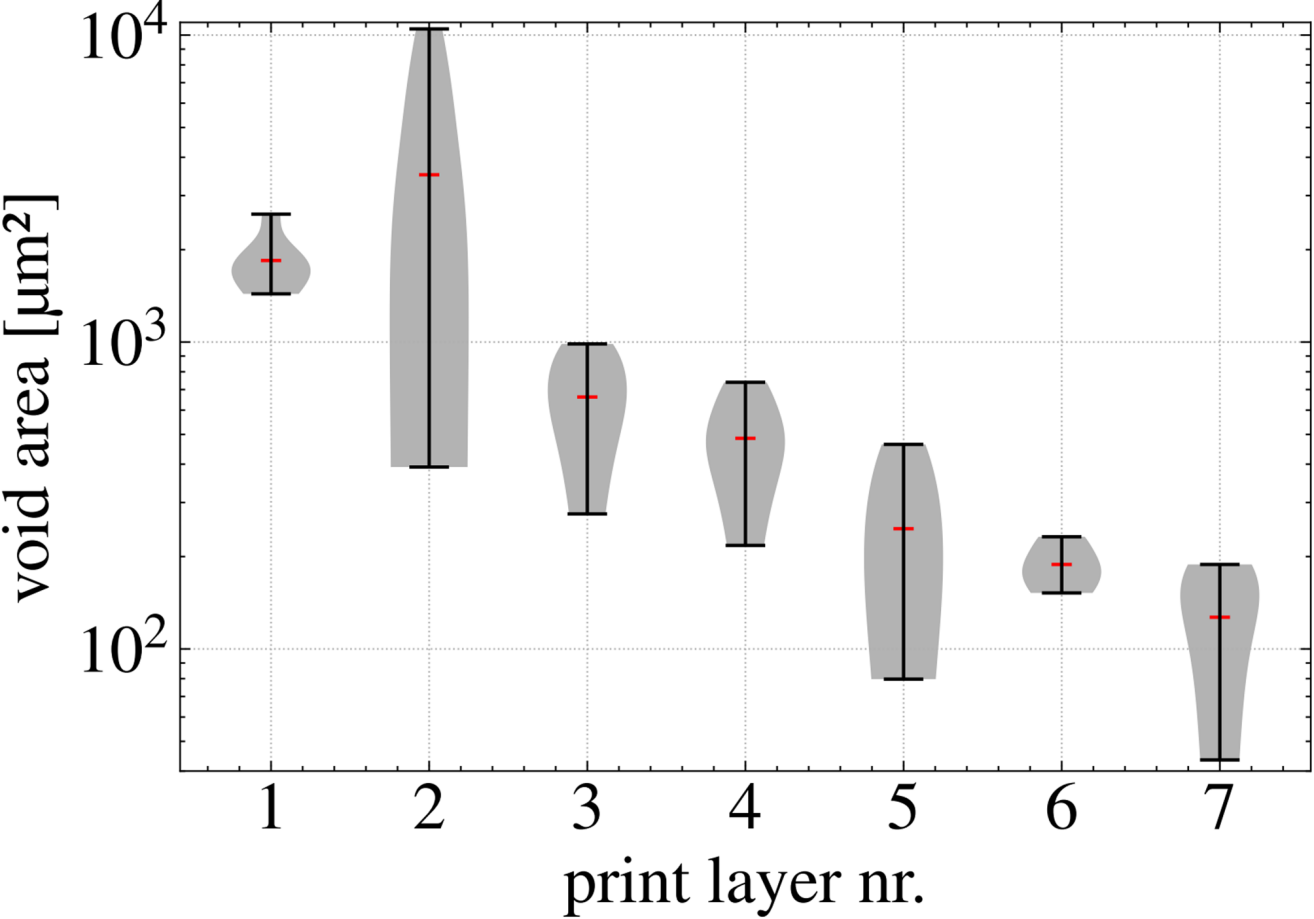
Void area measured in the cross-section of the H0 tensile specimen, shown as a function of the void’s position within the printed layer.

SEM micrographs and µCT scans were used to further investigate this hypothesis. SEM micrographs of the H90 and V90 specimens (Fig. 8A) show a more irregular fracture surface with a clearly recognizable layered structure (H90) and strand structure (V90) in the fracture pattern. Moreover, the H0 cross-section already reveals the presence of voids in the transition region between the first and the second layer, which is confirmed by µCT scans of H90 specimens (Fig. 8B). These voids are symptoms of reduced interlayer bonding^24,25^. Such voids decrease the bonding strength resulting in reduced UTS and elongation at break. Void area in dependence of its location in the printed layer is presented in Fig. 9. While relatively big voids a present in the second layer, the void size drops by at least one order of magnitude when the layer number and hence the distance to the print bed increases.

The relatively large voids observed in the lower layers may be attributed to a temperature drop from 200 °C to 180 °C following the deposition of the first layer, as observed in both H0 and H90 specimens. This temperature undershoot is likely due to the delayed response of the PID controller, resulting in filament being extruded at a lower temperature than intended. In addition, strong heat conduction into the print bed accelerated filament solidification, promoting void formation and reducing interlayer bonding quality. In contrast, the significant reduction in void size in higher layers suggests a decline in heat conduction with increasing distance from the bed surface. This slower cooling likely allowed for more gradual solidification and improved bonding. Notably, such height-dependent variations in pore structure are typically not reported under atmospheric conditions, where pore size is typically uniform across the part^26^.

To reduce hot-end thermal inertia and minimize temperature deviation in vacuum, the hot-end volume should be reduced, and control algorithms refined, e.g., by implementing model predictive temperature control^27^.

The V90 specimens exhibited a modest reduction in both elongation at break and Young’s modulus compared to the H0 specimens. This indicates a stiffer mechanical response, with failure occurring at lower strain and a slightly reduced ultimate tensile strength (UTS). In this print orientation, the specimens were fabricated vertically, thereby minimizing the thermal influence of the heated print bed. Under vacuum conditions, the absence of convective heat transfer slows down the filament cooling process, which in turn promotes improved interlayer bonding due to extended thermal retention. This enhanced bonding is a likely explanation for the increased stiffness observed in V90 specimens relative to the H90 configuration. Supporting this interpretation, micro-computed tomography (µCT) cross-sections of the V90 specimens revealed a more uniform and denser filament structure.

Despite the generally improved bonding, small voids were observed near the left boundary of the µCT cross-section. These voids were located at the interface between the outermost layer—printed at a slower speed of 5 mm/s—and the subsequent body layers printed at 10 mm/s. The occurrence of these voids is likely due to differential cooling behavior: the slower-printed outer layer had more time to cool and potentially shrink before the adjacent layers were deposited. As a result, the interfacial temperature was lower during bonding, which may have weakened adhesion locally and led to void formation. This illustrates how local cooling history and speed strongly affect interlayer quality, even in vacuum.

In addition, µCT scans were conducted to evaluate the porosity of the H90 and V90 specimens. The average porosity was determined to be 0.67% for H90 and 0.1% for V90. As shown in Fig. 8B, the voids in the H90 specimens were primarily located near the print bed surface, whereas in the V90 specimens they appeared adjacent to the outer perimeter. These porosity values are notably low and are typically associated with specimens printed in air at much higher extrusion temperatures (> 240 °C)^26^. This indicates that printing in a vacuum environment—by suppressing convective cooling—can facilitate the production of high-quality parts with minimal porosity, even at reduced processing temperatures.

Polymer rheology critically influences process stability and print quality in extrusion-based AM, including under vacuum. Although this study lacks direct rheological tests, literature offers key insights into how melt properties affect FFF outcomes in vacuum.

In extrusion processes, polymer melt viscosity governs nozzle flow, bonding, die swell, and filament stability As highlighted by Makay^28^, shear thinning and relaxation dynamics influence how well the filament conforms and bonds to underlying layers. Das et al.^29^ further emphasize that rheological properties govern the extent of polymer chain interdiffusion and welding—key mechanisms for mechanical integrity in printed parts.

The potential influence of vacuum on the rheological behavior of polymers is hypothesized in accordance with prior literature. Our experimental results suggest, that under vacuum, absence of convective cooling and thus altered thermal gradients can modify the effective shear and temperature fields experienced by the polymer. This may amplify the influence of rheological parameters such as complex viscosity, storage modulus, and relaxation time. The slower cooling under vacuum conditions could prolong the melt state of the deposited filament, potentially enhancing interlayer diffusion but also increasing the risk of deformation due to insufficient solidification.

While this study focuses on PLA as a model material, the underlying phenomena observed under high vacuum—such as reduced cooling rates and enhanced interlayer bonding—are expected to be even more critical for high-performance engineering polymers such as PEEK, PEKK and PEI. These materials exhibit a much steeper temperature–viscosity relationship than PLA, meaning that small variations in thermal history can lead to substantial differences in melt flow behavior, weld strength, and crystallization kinetics. In particular, for materials like PEEK, even minor cooling rate changes can significantly affect interlayer diffusion and bonding quality. Additionally, vacuum environments inherently reduce ambient convective heat transfer and external viscous drag, potentially leading to modified extrusion flow profiles and strand geometry. These effects may influence print fidelity, fusion quality, and dimensional accuracy, especially for space-relevant polymers processed near their thermal limits. However, practical challenges such as thermal management and dynamic behaviour of high-temperature hot ends, material outgassing, and thermal degradation must be addressed before extending vacuum-based FFF to aerospace-grade polymers. Future studies should therefore consider tailored process control strategies and dedicated hardware adaptations when applying these findings to advanced materials.

While printing of components included operation over several hours, long-term system stability, print-to-print variability, and contamination risks under extended vacuum operation were not systematically evaluated in this study. These factors are particularly relevant for in-space additive manufacturing, where maintenance opportunities are limited, and consistent part quality is critical. Future work will focus on quantifying system reliability through repeated fabrication cycles, monitoring changes in mechanical and thermal performance over time, and assessing potential contamination from outgassing or particulate build-up on surface of critical components such sensors and mirrors and measures that can be taken to prevent the contamination.

## Conclusions

This study demonstrated the effective operation of a fused filament fabrication (FFF) system under high-vacuum conditions, achieving stable printing at pressures as low as 5 × 10⁻⁴ mbar. The elimination of convective heat transfer significantly altered thermal dynamics, necessitating the adjustment of PID control parameters to maintain consistent hot-end temperatures. Thermal imaging confirmed improved thermal stability and higher nozzle temperatures in vacuum compared to atmospheric conditions.

Mechanical testing highlighted the importance of thermal history and interlayer bonding in determining mechanical performance under vacuum. V90 specimens exhibited enhanced stiffness and lower void content, attributed to slower cooling and improved thermal retention. In contrast, H90 specimens displayed reduced bonding quality, likely due to faster conductive cooling to the print bed. Although SEM and µCT analyses revealed void formation in the initial layers—likely resulting from PID temperature undershoot and accelerated solidification—the overall porosity of the specimens remained low.

These findings underscore the potential of vacuum-assisted FFF for improving print quality and reducing porosity, even at lower extrusion temperatures. However, they also highlight the sensitivity of inter layer bonding to localized thermal fluctuations and print speed variations. Achieving high-performance parts with minimized anisotropy in convection-free environments requires careful optimization of thermal management and process control.

To further advance vacuum-based FFF, future work should focus on reducing the thermal mass of the hot end, improving control algorithms for dynamic temperature stability and implementing real-time thermal monitoring (e.g., infrared sensing). Additionally, controlled cooling strategies will be essential to ensure uniform layer solidification and prevent localized thermal inconsistencies. Moreover, rheological profiling should be performed (e.g., via oscillatory shear or capillary rheometry) to predict and optimize extrusion behavior under vacuum. In addition, developing or adapting existing rheological models to account for pressure-dependent heat transfer and melt dynamics could help adjust process control and material selection for vacuum-based FFF.

## Supplementary Information

Below is the link to the electronic supplementary material.

Supplementary Material 1

## Data Availability

Original data supporting the conclusions of this study were generated using our custom made vacuum FFF equipment. Derived data that supports the findings of this research can be obtained upon request from the corresponding author.
