# Supplementary material for: Fused filament fabrication of thermoplastics in high vacuum without convective heat transfer
Source: Sci Rep. 2025 Jul 28;15:27497. doi: 10.1038/s41598-025-13181-2 (PMC12304254; doi:10.1038/s41598-025-13181-2)
Supplement: Supplementary file 1 — Supplementary Material 1 [file 41598_2025_13181_MOESM1_ESM.docx]

**Supplementary Materials**


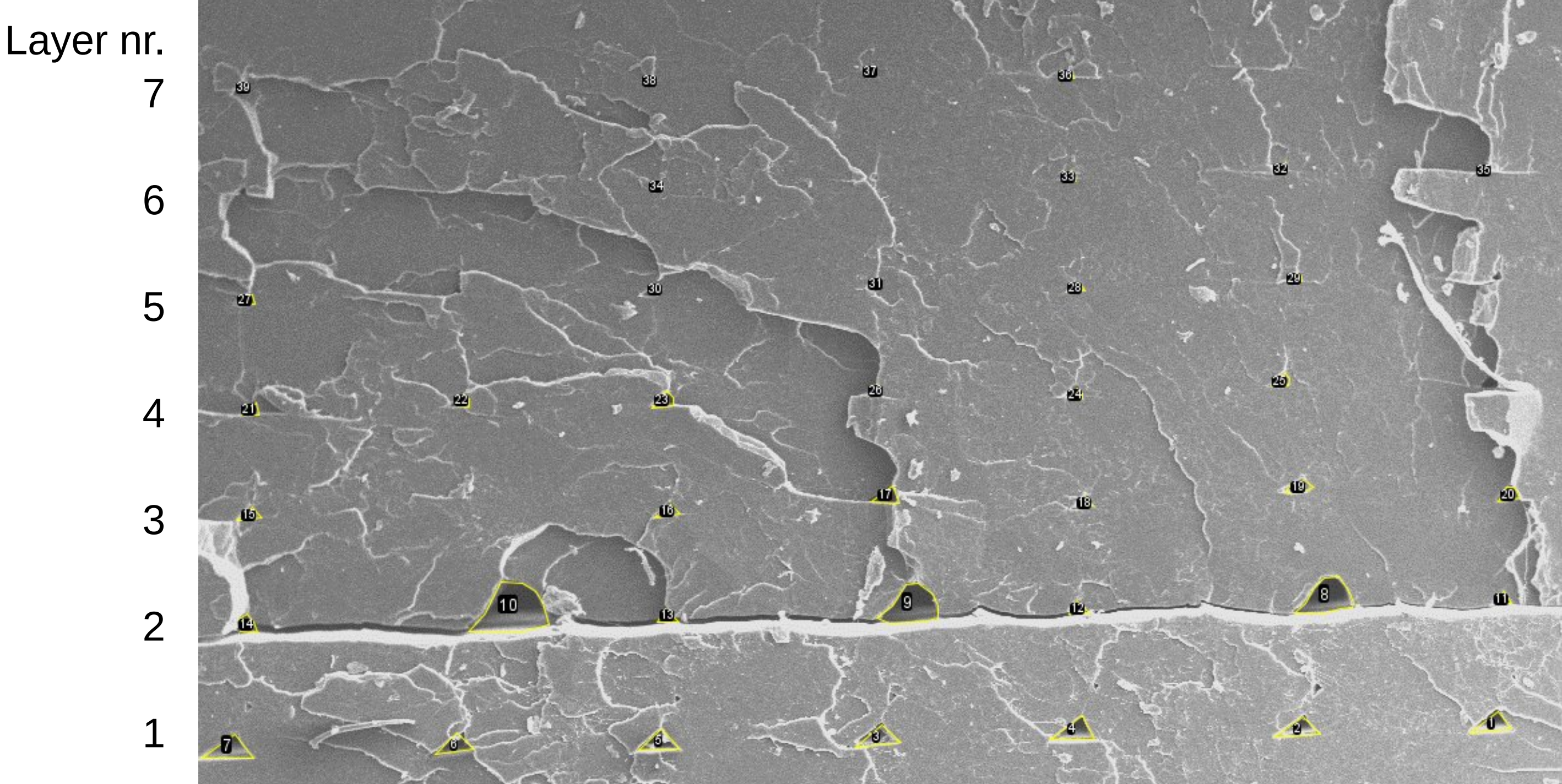


Figure S1: Quantitative analysis of void size in the SEM micrographs of cross-section of H0 tensile bars using ImageJ software. Here the layer which has the shortest distance to the print bed is labelled 1.
